# Meroterpenes from Endophytic Fungus A1 of Mangrove Plant *Scyphiphora hydrophyllacea*

**DOI:** 10.3390/md10091993

**Published:** 2012-09-17

**Authors:** Wen-Li Mei, Bo Zheng, You-Xing Zhao, Hui-Ming Zhong, Xun-Li Wu Chen, Yan-Bo Zeng, Wen-Hua Dong, Jiu-Li Huang, Peter Proksch, Hao-Fu Dai

**Affiliations:** 1 Key Laboratory of Biology and Genetic Resources of Tropical Crops, Ministry of Agriculture, Institute of Tropical Bioscience and Biotechnology, Chinese Academy of Tropical Agriculture Sciences, Haikou 571101, China; Email: meiwenli@itbb.org.cn (W.-L.M.); zhengbofootball@163.com (B.Z.); zhaoyx1011@163.com (Y.-X.Z.); xyc9530@163.com (X.-L.W.C.); zengyanbo@163.com (Y.-B.Z.); dwh962@163.com (W.-H.D.); huangjiuli999@163.com (J.-L.H.); 2 College of Chemistry and Molecular Engineering, Qingdao University of Science and Technology, Qingdao 266042, China; Email: zhonghuimin@qust.edu.cn; 3 Institute of Pharmaceutical Biology and Biotechnology, Heinrich-Heine-Universitaet Duesseldorf, Duesseldorf 40225, Germany; Email: Proksch@uni-duesseldorf.de

**Keywords:** marine endophyte, *Scyphiphora hydrophyllacea*, meroterpenes, *Guignardia* sp., MRSA, *Staphylococcus aureus*

## Abstract

Four new meroterpenes, guignardones F–I (**1**–**4**), together with two known compounds guignardones A (**5**) and B (**6**) were isolated from the endophytic fungus A1 of the mangrove plant *Scyphiphora hydrophyllacea*. Their structures and relative configurations were elucidated by spectroscopic data and single-crystal X-ray crystallography. A possible biogenetic pathway of compounds **1**–**6 **was also proposed. All compounds were evaluated for inhibitory activity against methicillin-resistant *Staphylococcus aureus* (MRSA) and *Staphylococcus aureus*.

## 1. Introduction

**Figure 1:**
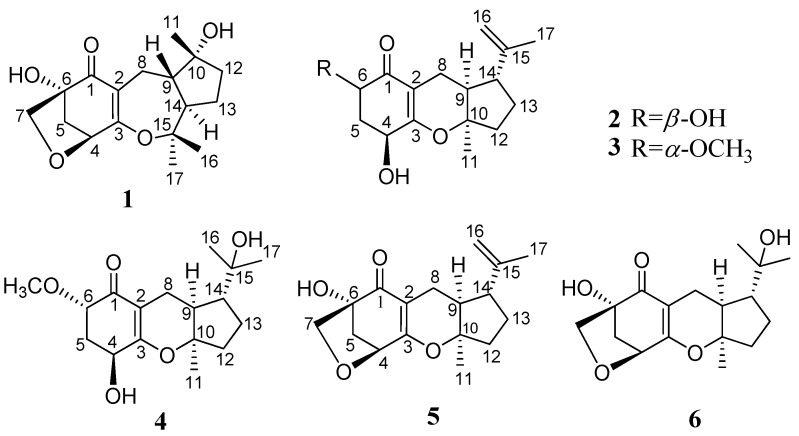
The structures of compounds **1**–**6**.

Marine-derived fungi have recently become a research focus as one of the richest sources of new and bioactive secondary metabolites in the marine environment [1,2]. The mangrove plant *Scyphiphora hydrophyllacea* Gaertn. F. (Rubiaceae) growing in tropical and subtropical intertidal habitats is a rich source of new iridoids, which have been found to possess cytotoxic activity [3,4,5,6]. The metabolites produced by endophytic fungi from *S. hydrophyllacea* collected in Hainan attracted our attention [7], a new fatty acid glycoside and two new meroterpenes have been found from the fungus A1 of *S. hydrophyllacea* [8,9]. Further phytochemical investigation of the secondary metabolites from the culture broth of the fungus A1 led to the isolation of four new meroterpenes, guignardones F–I (**1**–**4**) (Figure 1), and two known compounds, guignardones A (**5**) and B (**6**) [10]. This paper reports the isolation and structural elucidation, as well as biological activities and plausible biogenetic pathway of these compounds.

## 2. Results and Discussion

**Figure 2:**
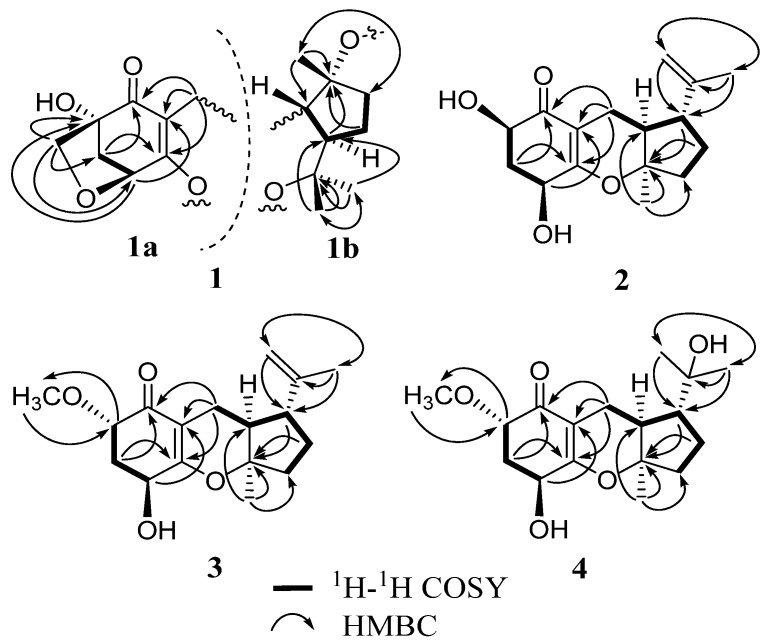
^1^H-^1^H COSY and key HMBC correlations of **1**–**4**.

**Chart 1:**
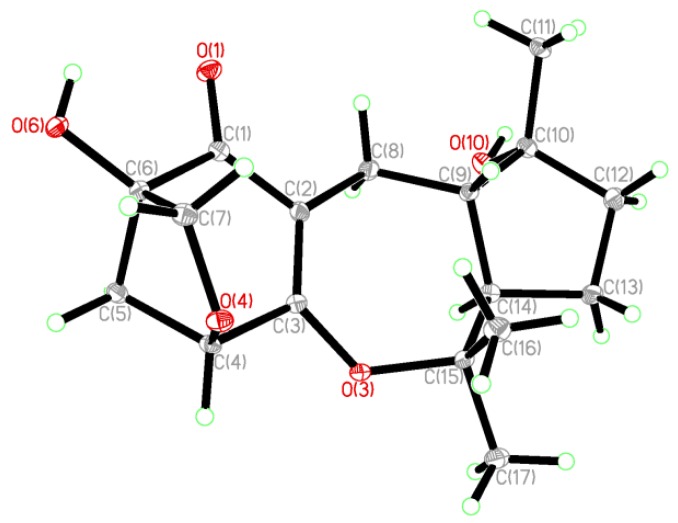
The ORTEP view of compound **1**.

Guignardone F (**1**) was isolated as colorless needle crystals, and possessed a molecular formula of C_17_H_24_O_5 _as established by HREIMS (*m/z* 308.1625 [M]^+^), indicating six degrees of unsaturation. The IR spectrum displayed hydroxyl (3463 cm^−1^), conjugated carbonyl (1657 cm^−1^) and double bond (1606 cm^−1^) absorptions. In the ^13^C NMR and DEPT spectra (Table 1), a total of 17 carbon resonances were found and classified into three methyls, five methylenes (one oxygenated), three methines (one oxygenated), and six quaternary carbons (one α,β-unsaturated carbonyl unit, three oxygenated). In the HMBC spectrum (Figure 2), the correlations from H_2_-8 (δ_H_ 3.03, 1.92) to C-1 (δ_C_ 200.2), C-2 (δ_C_ 114.7), and C-3 (δ_C_ 173.9), from H-4 (δ_H_ 4.46) to C-2 and C-6 (δ_C_ 81.8), from H_2_-5 (δ_H_ 2.31, 1.91) to C-1 and C-3, and from H_2_-7 (δ_H_ 3.80, 3.45) to C-4 (δ_C_ 81.3), C-5 (δ_C_ 42.5), and C-6 were found and led to the establishment of partial structure **1a**, which was identical to that of guignardone B (**6**) [10]. In the ^1^H-^1^H COSY spectrum, a proton spin system (H-8/H-9/H-14/H-13/H-12) was found as drawn with bond lines in Figure 2. In addition, the HMBC cross-peaks from H_3_-11 (δ_H_ 1.35) to C-9 (δ_C_ 46.1), C-10 (δ_C_ 80.5), and C-12 (δ_C_ 39.3), and from both H_3_-16 (δ_H_ 1.17) and H_3_-17 (δ_H_ 1.45) to C-14 (δ_C_ 54.1) and C-15 (δ_C_ 86.9) determined the establishment of substructure **1b**. Careful comparison of the ^13^C NMR data of **1** with those of **6**, the upfield shift of C-10 (Δ −10.2 ppm) and the downfield shift of C-15 (Δ +14.0 ppm) were observed. So it was proposed that C-3 connected with C-15 via an ether bond to form a seven-membered ring in **1**, instead of the C-3 connected with C-10 via an ether bond to form a six-membered ring in **6**. This also gave a good explanation for the obvious difference (Δ 10.8 ppm) of chemical shifts between the 16-CH_3_ and the 17-CH_3_, since their stereochemistry circumstance was quite different in the structure of **1**. Thus, the gross structure of **1** was elucidated and further confirmed by X-ray crystallography which also established its relative configuration (Chart 1). Therefore, the structure of **1** was established as shown in Figure 1, named Guignardone F (**1**).

**Table 1:** ^13^C NMR data (100 MHz, CDCl_3_) for **1**–**4**.

position	1	2	3	4
1	200.2 s	198.7 s	194.9 s	195.0 s
2	114.7 s	105.0 s	105.8 s	105.5 s
3	173.9 s	167.1 s	167.7 s	168.5 s
4	81.3 d	66.7 d	65.7 d	65.7 d
5	42.5 t	36.9 t	34.5 t	34.6 t
6	81.8 s	67.1 d	79.1 d	79.0 d
7	69.3 t			
8	21.9 t	16.2 t	16.1 t	18.7 t
9	46.1 d	43.3 d	43.2 d	41.2 d
10	80.5 s	88.2 s	87.7 s	88.9 s
11	26.5 q	23.5 q	22.3 q	22.1 q
12	39.3 t	37.7 t	37.4 t	38.3 t
13	25.9 t	27.0 t	26.9 t	24.5 t
14	54.1 d	48.7 d	48.9 d	51.1 d
15	86.9 s	145.4 s	145.4 s	72.9 s
16	19.2 q	111.3 t	111.2 t	28.6 q
17	30.0 q	19.2 q	19.2 q	27.5 q
OCH_3_			58.3 q	58.3 q

**Figure 3:**
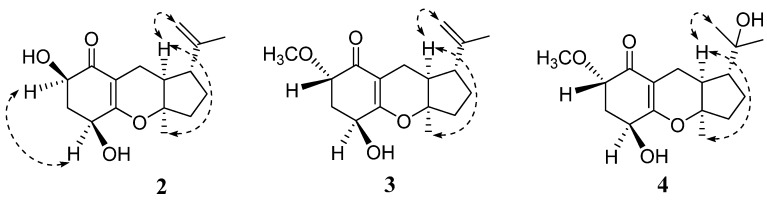
Selected ROESY correlations of **2**–**4**.

Guignardone G (**2**) was obtained as a colorless oil. Its molecular formula was determined as C_16_H_22_O_4_ from [M]^+^ ion peak at *m/z* 278.1513 in the HREIMS, with six degrees of unsaturation. IR absorptions implied the presence of hydroxyl (3440 cm^−1^), conjugated carbonyl (1653 cm^−1^) and double bond (1622 cm^−1^) groups. The ^13^C NMR and DEPT spectra (Table 1) revealed 16 carbon signals, including one α,β-unsaturated carbonyl group, one terminal double bond, one oxygenated quaternary carbon, four methines (two oxygenated), four methylenes, and two methyls. Apart from one carbonyl and two double bonds, the remaining degrees of unsaturation indicated that **2** possessed a tricyclic system. Detailed analysis of its ^1^H NMR and ^13^C NMR spectra showed that compound **2** was analogous to guignardone A (**5**) [10], except for the absence of a methylene (C-7), a quaternary carbon (C-6) in **5** and the presence of a methine (C-6) in **2**. In addition, the ^13^C NMR signals of C-4 upshifted from δ_C_ 78.5 to δ_C_ 66.7 and C-5 from δ_C_ 44.0 to δ_C_ 36.9. Base on the above evidence, it was supposed that the C-7 of **5** was degraded and the corresponding ring was broken to form the structure of **2**, which was confirmed by 2D NMR experiments. The relative configuration of **2** was assigned by a ROESY experiment and comparison with **5**. H-9 (δ_H_ 1.95) showed correlations to both H_3_-11 (δ_H_ 1.32) and H_2_-16 (δ_H_ 4.64), which indicated that when H-9 and CH_3_-11 possess α-orientation, H-14 (δ_H_ 2.27) will possess β*-*orientation, as in accordance with the relative configuration of **5 **(Figure 3). The α-orientation of H-4 (δ_H_ 4.35) was also tentatively established as same as that of **5 **for the biogenetic pathway consideration. H-6 (δ_H_ 4.48, dd, *J* = 11.9, 5.4 Hz) exhibited ROESY correlation with H-4, and the large vicinal coupling constant (*J* = 11.9 Hz) established the pseudoaxial position for H-6. So H-6 was established as α-oriented. Thus, the structure of **2** was established and named guignardone G.

Guignardone H (**3**) was isolated as a colorless oil. The HRTOFMS of **3** showed a pseudo-molecular ion peak at *m/z* 293.1745 [M + H]^+^ corresponding to the molecular formula C_17_H_24_O_4_, implying six degrees of unsaturation. The ^1^H NMR and ^13^C NMR spectra of **3 **(Table 1 and Table 2) were strikingly similar to those of **2**, with the only obvious difference being the presence of a methoxy (δ_H_ 3.47, 3H, s; δ_C_ 58.3) group in **3**. The HMBC correlations of the methoxy protons to C-6 (δ_C_ 79.1) and from H-6 (δ_H_ 3.70) to the methoxy carbon indicated that the methoxy group located at C-6. β-orientations of OH-4 and H-14 and α-orientations of H-9 and CH_3_-11 were established by ROESY correlations and comparison with **2 **and proved to be the same as **2**. There was no ROESY correlation between H-6 (δ_H_ 3.70, dd, *J* = 6.8, 3.6 Hz) and H-4 (δ_H_ 4.24), and the proton coupling constants between H-6 and H_2_-5 (δ_H_ 2.35, 2.23) were 6.8 and 3.6 Hz, differed from those of H-6 in **2**. Hence, H-6 was deduced to be β-oriented. Thus, the structure of **3** was established as shown (Figure 1) and named guignardone H.

**Table 2:** ^1^H NMR data (400 MHz, CDCl_3_) for **1**–**4**.

position	1	2	3	4
4	4.46, d (5.2)	4.35, br t (2.8)	4.24, br s	4.26, br s
5	2.31, m	2.53, dq (13.4, 5.4)	2.35, m	2.38, dt (13.6, 4.2)
	1.91, m	2.01, m	2.23, m	2.23, m
6		4.48, dd (11.9, 5.4)	3.70, dd (6.8, 3.6)	3.71, dd (7.0, 3.6)
7	3.80, d (8.0)			
	3.45, d (8.0)			
8	3.03, dd (15.0, 3.2)	2.32, m	2.33, d (17.3)	2.62, d (17.6)
	1.92, m	2.28, m	2.15, m	2.26, m
9	1.40, dd (12.0, 3.2)	1.95, m	1.94, m	2.04, m
11	1.35, s, 3H	1.32, s, 3H	1.33, s, 3H	1.33, s, 3H
12	1.76, m, 2H	2.10, m; 1.81, m	2.13, m; 1.79, m	2.01, m; 1.59, m
13	1.95, m; 1.22, m	1.94, m; 1.57, m	1.91, m; 1.53, m	1.79, m; 1.58, m
14	2.33, m	2.27, m	2.17, m	1.58, m
16	1.17, s, 3H	4.73, m	4.72, m	1.20, s, 3H
		4.64, m	4.62, m	
17	1.45, s, 3H	1.66, s, 3H	1.65, s, 3H	1.18, s, 3H
OCH_3_			3.47, s, 3H	3.47, s, 3H

Guignardone I (**4**) was obtained as a colorless oil. The molecular formula was deduced as C_17_H_26_O_5_ on the basis of its HREIMS ion peak at *m/z* 310.1783 [M]^+^ with five degrees of unsaturation. The ^1^H NMR and ^13^C NMR data (Table 1 and Table 2) of **4** closely resembled those of **3**, except for the disappearance of an exocyclic double bond and the presence of an oxygenated quarternary carbon (C-15, δ_C_ 72.9) and a methyl group (C-16, δ_C_ 28.6) in **4**. Therefore, it was supposed that compound **4 **may derived from **3** by hydroxylation at the exocyclic double bond, which was confirmed by the HMBC correlations of H_3_-16 (δ_H_ 1.20) with C-14 (δ_C_ 51.1), C-15 (δ_C_ 72.9), and C-17 (δ_C_ 27.5). The ROESY correlations and chemical shifts at chiral centers of **4 **were similar to those of **3**, which showed the same relative configurations at C-4, C-6, C-9, C-10, and C-14.

**Scheme 1:**
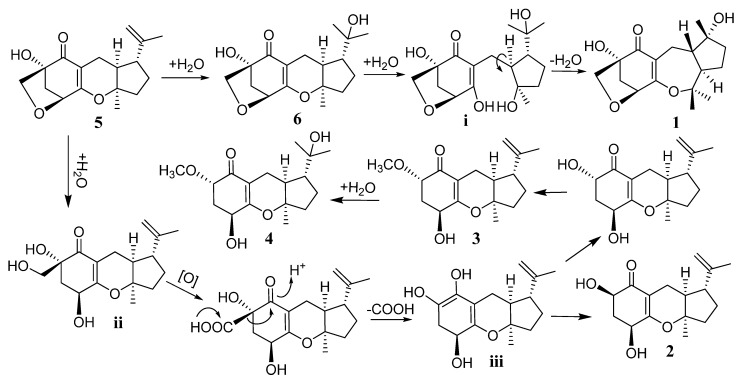
Plausible biosynthetic pathway of **1**–**6**.

A possible biogenetic pathway of compounds **1**–**6 **was proposed as shown in Scheme 1. Guignardone A (**5**), a major product isolated in this experiment, was taken as the biogenetic precursor. Two ether bonds were suitable for the enzymatic opening that resulted in a 3,10-dihydroxy intermediate (i) and a 4,7-dihydroxy intermediate (ii). The five-membered ring and the connected isopropanol group of intermediate **i **could turn upside-down around the bond C-8–C-9 to generate **1**. The intermediate **ii** underwent an oxidation and decarboxylation to give an intermediate (iii). Afterward, the intermediate (iii) converted to its C-1 ketone isomer. This assumption reasonably accounted for the simultaneous occurrences of β-orientation of OH-6 in **2** and α-orientation of OCH_3_ in **3**.

All compounds were tested for antibacterial activities against methicillin-resistant *Staphylococcus aureus* (MRSA) and *Staphylococcus aureus*. Guignardone I (**4**) exhibited inhibition zones of 9.0 and 11.0 mm in diameter (the diameter of sterile filter paper discs was 6 mm) toward MRSA and *S. aureus* at 65 µM, respectively. Guignardone B (**6**) gave an inhibition zone of 8.0 mm against MRSA at 65 µM. The other compounds have not show any antibacterial action under the same conditions. 10 µL 0.08 mg mL^−1^ kanamycin sulfate was used as positive control, for which the diameter of the inhibition zone was 30 mm. 

## 3. Experimental Section

### 3.1. General Experimental Procedures

Optical rotations were taken on a Rudolph Autopol Ш. IR spectra were measured on a Nicolet 380 FT-IR instrument with KBr pellets. NMR spectra were recorded on a Bruker AM-400 spectrometer at 400 MHz for ^1^H NMR and at 100 MHz for ^13^C NMR using TMS as an internal standard. MS spectra were obtained on a VG Auto Spec-3000 mass spectrometer (VG, Manchester, UK). Column chromatography was performed on silica gel (200–300 mesh; Qingdao Marine Chemical Inc., Qingdao, People’s Republic of China). Sephadex LH-20 for chromatography was purchased from Merck (Germany). TLC was performed with silica gel GF254 (Marine Chemical Inc, Qingdao, China), and spots were visualized by heating silica gel plates sprayed with 10% H_2_SO_4_ in EtOH.

### 3.2. Fungal Material and Fermentation

The marine-derived fungus A1 was isolated from the leaves of mangrove plant *Scyphiphora hydrophyllacea* Gaertn. F. (Rubiaceae) (No. SH20081205), which were collected in Wenchang County in Hainan Province (China) in December 2008. The strain, which was identified as a *Guignardia* sp. based on the ITS sequences, was deposited in the Institute of Tropical Bioscience and Biotechnology, Chinese Academy of Tropical Agricultural Sciences, Haikou, China, and maintained on potato dextrose agar (PDA) slant at 4 °C. The marine-derived fungus A1 was grown on PDA at room temperature for 5 days. Three pieces of mycelial agar plugs (0.5 × 0.5 cm^2^) were inoculated into 1 L Erlenmeyer flasks containing 300 mL potato dextrose broth. The cultivation was shaken at 120 rpm at room temperature for 7 days, and then kept in still at room temperature for 45 days.

### 3.3. Extraction and Isolation

The culture broth (130 L) was filtered to give the filtrate and mycelia. The filtrate was evaporated *in vacuo* to small volume and then partitioned in succession between H_2_O and petroleum ether, EtOAc, *n*-Butanol. The EtOAc solution was evaporated under reduced pressure to give a crude extract (10.5 g), which was separated into 11 fractions (Fr.1–Fr.11) on a silica-gel column using a step gradient elution of CHCl_3_/MeOH (1:0→0:1). The Fr.1 (732 mg) was firstly subjected to Sephadex LH-20 (CHCl_3_/MeOH, 1:1) CC, then subjected to silica gel CC (Pet/EtOAc 12:1, Pet/acetone 10:1) repeatedly to give **5** (80.6 mg) and **3** (12.5 mg). The Fr.3 (583 mg) was subjected to silica gel CC (Pet/Acetone 8:1 to 1:1, Pet/EtOAc 6:1, CHCl_3_/EtOAc 6:1) repeatedly, and further purified by Sephadex LH-20 (CHCl_3_/MeOH, 1:1) CC to give **1** (11.3 mg), **2** (8.5 mg), and **6** (6.2 mg). The Fr.4 (468 mg) was purified by silica gel CC (Pet/Acetone 6:1 to 1:1, Pet/EtOAc 4:1, CHCl_3_/MeOH 40:1) repeatedly to give **4** (9.2 mg). 

Guignardone F (**1**): Colorless needle crystals; [α]^32^_D_−42 (*c* 0.20, MeOH); IR (KBr) ν_max_ 3463, 3285, 2973, 2942, 2877, 1657, 1606, 1455, 1375, 1232, 1186, 1131, 1092, 1021 cm^−1^; ^1^H and ^13^C NMR, see Table 1 and Table 2; HREIMS *m/z* 308.1625 [M]^+^ (calcd for C_17_H_24_O_5_, 308.1624). 

Guignardone G (**2**): Colorless oil; [α]^32^_D_ +3.5 (*c* 0.32, MeOH); IR (KBr) ν_max_ 3440, 2955, 2925, 2854, 1653, 1622, 1458, 1378, 1167, 1099, 1059 cm^−1^; ^1^H and ^13^C NMR, see Table 1 and Table 2; HREIMS *m/z* 278.1513 [M]^+^ (calcd for C_16_H_22_O_4_, 278.1518).

Guignardone H (**3**): Colorless oil; [α]^32^_D_ +8.9 (*c* 0.34, MeOH); IR (KBr) ν_max_ 3439, 2927, 1658, 1616, 1442, 1394, 1378, 1168, 1077, 1019, 890 cm^−1^; ^1^H and ^13^C NMR, see Table 1 and Table 2; HRTOFMS *m/z* 293.1745 [M + H]^+^ (calcd for C_17_H_25_O_4_, 293.1752).

Guignardone I (**4**): Colorless oil; [α]^32^_D_ −32 (*c* 0.24, MeOH); IR (KBr) ν_max_ 3440, 2968, 2930, 1654, 1613, 1458, 1396, 1379, 1364, 1281, 1164, 1131, 1077, 1020, 887 cm^−1^; ^1^H and ^13^C NMR, see Table 1 and Table 2; HREIMS *m/z* 310.1783 [M]^+^ (calcd for C_17_H_26_O_5_, 310.1780).

Crystallographic data for Guignardone F (**1**): C_17_H_24_O_5_; MW = 308.36; Monoclinic, space group *C2*; 9.7072 (17) Å, *b* =9.7382 (16) Å, *c* = 16.752 (3) Å, α =β =γ = 90°, *V* = 1583.6 (5) Å^3^, *Z* = 4, *d* = 1.293 g/cm^3^, crystal dimensions 0.08 × 0.20 × 0.70 mm^3^.

### 3.4. Antibacterial Activity

Compounds **1**–**6** were tested for antibacterial activity against *S*. *aureus* and MRSA strains (obtained from the Food and Drug Administration of Hainan Province, Haikou, China) using the filter paper disc agar diffusion method [11]. The strains were cultured using nutrient agar. Fifty microliters (28 mM) of the compound were impregnated on sterile filter paper discs (6-mm diameter), and then, aseptically applied to the surface of the agar plates. Ten microliters (0.08 mg/mL) of kanamycin sulfate were used as positive control. Then the diameters of inhibition zones were measured after 24 h incubation at room temperature. Experiments were done in triplicate, and the results presented as mean values of the three measurements.

## 4. Conclusions

Four new meroterpenes, guignardones F–I (**1–4**), together with two known compounds guignardones A (5) and B (6), were isolated from the endophytic fungus A1 *Guignardia* sp. of mangrove plant *Scyphiphora hydrophyllacea*. The plane structure of guignardone G was same as coibanol A, one of the three tricyclic meroterpenes isolated from an endophytic fungus *Pycnoporus sanguineus*. The H-9 and CH_3_-11 possess α-orientations in compounds **1–6**, different from the β*-*orientation in coibanols A–C [12]. The plane structure of guignardone H was same as tricycloalternarene F, another tricyclic meroterpene isolated from an endophytic fungus *Guignarda mangiferae*, the stereochemistry of which has not been clarified [13]. Guignardones A (5) and B (6) with an additional tetrahydrofuran ring in their structure have been isolated from a fungus *Guignardia mangiferae *associated with normal *Ilex cornuta *leaves [10]. Especially, compound **1** possessed a novel structure, beside an additional tetrahydrofuran ring, the six-membered ring possessing an oxygen atom was changed to a seven-membered ring. Antibacterial tests demonstrated that guignardone I (**4**) showed modest inhibitory effects on *Staphylococcus aureus* and MRSA, diameters of inhibition zones of which were 9.0 and 11.0 mm, respectively. Guignardone B (**6**) exhibited weak antibacterial activity against MRSA with a diameter of inhibition zones of 8.0 mm.
